# CRISPR-Cas9 Gene Editing in *Aspergillus*: From Pathogenesis to Metabolic Engineering

**DOI:** 10.3390/biology15010053

**Published:** 2025-12-28

**Authors:** Danni Hu, Ruoyu Zhao, Yingxu Lin, Chunmiao Jiang

**Affiliations:** Jiangxi Key Laboratory of Natural Microbial Drug Research, College of Life Sciences, Jiangxi Science & Technology Normal University, Nanchang 330013, China; hudanni0818@gmail.com (D.H.); a17858954618@163.com (R.Z.); 15087187287@163.com (Y.L.)

**Keywords:** *Aspergillus* species, genome editing technology, CRISPR-Cas9, protospacer adjacent motif, metabolic engineering

## Abstract

The genus *Aspergillus* comprises over 600 species of filamentous fungi. This genus significantly impacts human health, food fermentation, and industrial biotechnology. With the in-depth research and applications of *Aspergillus* species in many fields, the establishment of efficient gene editing technologies is crucial for functional genomics studies and cell factory development. The clustered regularly interspaced short palindromic repeats and associated protein (CRISPR-Cas9) system, as a newly developed and powerful genome editing tool, has demonstrated exceptional potential for precise genetic modifications in various *Aspergillus* species. The continuous advancement of CRISPR-Cas9 technology has enabled precise gene editing and modification in both pathogenic and industrial *Aspergillus* strains, thereby driving innovations in pathogenicity attenuation, metabolic engineering, and functional genomics. Therefore, this review provides a concise overview of the CRISPR-Cas9 system, detailing its composition, working mechanism, and key functional features such as the role of the Cas9 protein and the protospacer adjacent motifs (PAMs). Subsequently, we focus on the transformative applications of CRISPR-Cas9 in *Aspergillus* species, discussing its pivotal roles in elucidating pathogenic mechanisms, disrupting mycotoxin biosynthesis, and employing metabolic engineering to enhance the production of industrial enzymes, organic acids, and valuable natural products. Finally, we discuss future challenges and promising opportunities for applying CRISPR-Cas9 technology to advance the industrial biotechnology of *Aspergillus* species.

## Simple Summary

*Aspergillus* molds play dual roles in industry and as opportunistic pathogens. Understanding and optimizing their functions requires precise genome editing. The CRISPR–Cas9 system, adapted from bacterial immunity, has become a key tool for targeted genetic modification in *Aspergillus*. It enables research into disease mechanisms, reduction in mycotoxin production, and the engineering of strains for industrial applications such as enzyme and metabolite production. This review outlines the principles of CRISPR–Cas9, highlights its applications in *Aspergillus* species, and discusses future challenges in developing safer, more efficient fungal strains.

## 1. Introduction

Filamentous fungi are indispensable to medicine, agriculture, and industry. Among them, the genus *Aspergillus* represents one of the most studied and economically important fungal groups. Species within this genus are commonly found in environments such as soil, seeds, grains, and decaying vegetation. To date, more than 600 *Aspergillus* species have been identified based on morphological, physiological, and phylogenetic characteristics (https://www.catalogueoflife.org/, accessed on 15 June 2025) [1,2]. Although these fungi function primarily as terrestrial decomposers and can serve as pathogens of plants and humans, they are also of indispensable value in agricultural, food, and pharmaceutical applications.

**Figure 1:**
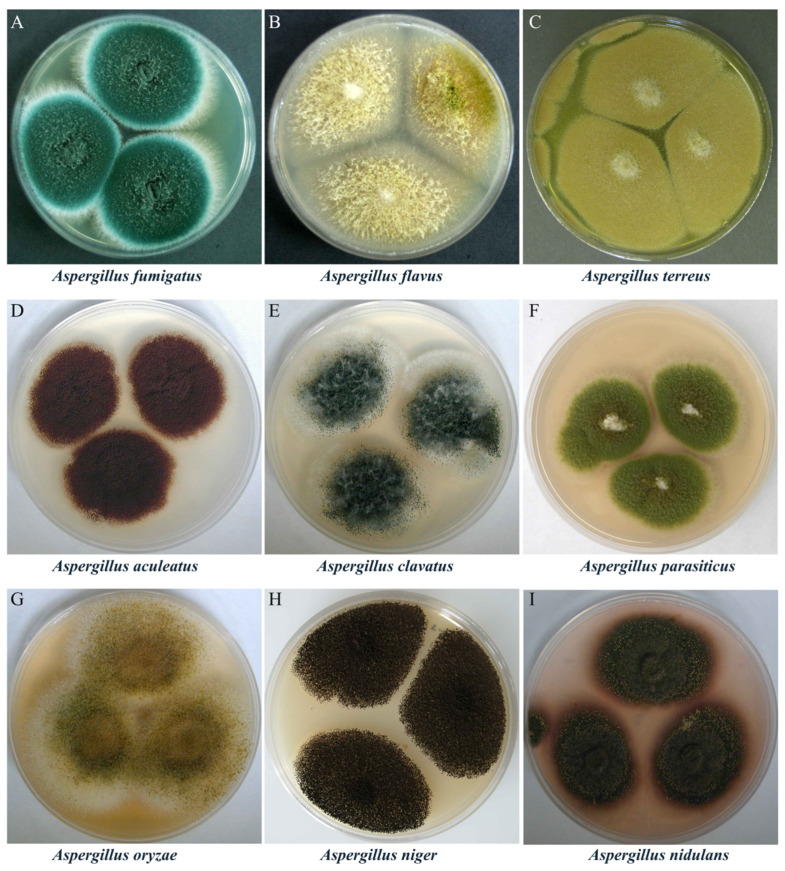
Colony morphology of representative *Aspergillus* species. Images reproduced from https://fungi.myspecies.info/all-fungi/aspergillus, licensed under CC BY-NC; corresponding images are also available in Baniya, S. *Aspergillus*: Morphology, Clinical Features, and Lab Diagnosis. Mycology. Available online: https://microbeonline.com/aspergillus-morphology-clinical-features-and-lab-diagnosis/#Aspergillus_glaucus (accessed on 20 June 2025). The panels show the typical macroscopic appearance of each species: (**A**) *Aspergillus fumigatus* (blue-green, powdery); (**B**) *Aspergillus flavus* (yellow-green, velvety); (**C**) *Aspergillus terreus* (yellow-brown to cinnamon); (**D**) *Aspergillus aculeatus* (brown); (**E**) *Aspergillus clavatus* (center black with a white periphery and abundant mycelium); (**F**) *Aspergillus parasiticus* (largely green with a white central area); (**G**) *Aspergillus oryzae* (yellow-green with a white margin); (**H**) *Aspergillus niger* (black, powdery); (**I**) *Aspergillus nidulans* (earthy brown).

Notable *Aspergillus* species of industrial, environmental, and clinical relevance include *Aspergillus fumigatus, Aspergillus flavus, Aspergillus terreus, Aspergillus nidulans, Aspergillus niger, Aspergillus aculeatus, and Aspergillus oryzae* (Figure 1). This genus exhibits a dual nature in its relationship with humans. While many species are indispensable in food fermentation, enzyme production, and pharmaceutical manufacturing, others pose serious threats to human health due to their production of harmful mycotoxins, including aflatoxins [3,4,5]. These risks are primarily associated with pathogenic species such as *A. flavus*, *A. fumigatus*, and *A. terreus* (Figure 1A–C). For instance, *A. fumigatus* is a saprophytic, filamentous fungus that is crucial for carbon and nitrogen recycling in nature, yet it is also a causative agent of several pulmonary diseases in humans, birds, and other mammals [6]. *A. flavus* is the second most common cause of invasive aspergillosis (after *A. fumigatus*) and the leading cause of superficial *Aspergillus* infections. Moreover, it produces aflatoxin B1, one of the most potent hepatocarcinogenic natural compounds known, along with other toxic metabolites such as cyclopiazonic acid, ustiloxin B, and aflatrem [7]. These examples underscore the significant health risks posed by pathogenic *Aspergillus* species.

Conversely, many *Aspergillus* species (particularly *A. oryzae*, *A. niger*, and *A. nidulans*) (Figure 1G–I) serve as cornerstones of industrial biotechnology due to their exceptional metabolic versatility. Their efficient secretion systems and adaptability to low-cost substrates have enabled the engineering of these fungi for large-scale production of enzymes, organic acids, and secondary metabolites [8,9]. Owing to their robust metabolic and secretory capabilities, these species are also widely used to produce valuable natural products, as well as homologous and heterologous proteins [10,11]. For example, the Generally Regarded as Safe (GRAS) species *A. oryzae* produces diverse enzymes and beneficial secondary metabolites, enabling its extensive applications in food fermentation (e.g., soy sauce, miso) and industrial processes [12,13]. Its high secretory capacity for hydrolytic enzymes has also established *A. oryzae* as a key cell factory for the production of bioactive secondary metabolites and industrial enzyme preparations [14,15,16]. Similarly, *A. niger* is known for its ability to produce commercially important enzymes, organic acids, and secondary metabolites, particularly glucoamylase, a key enzyme for starch hydrolysis in the food and beverage industries [17]. A recent study reports that *A. niger* produces a β-galactosidase with high lactose hydrolysis efficiency (>90%) and shows potential for prebiotics synthesis, achieving a 7% conversion yield [18]. This species also produces other enzymes such as amylases, proteases, and cellulases, which have applications in the production of biofuels, animal feed, and other biotechnological processes. Notably, *A. niger* has been employed for over a century in the industrial production of organic acids and accounts for approximately 99% of the global citric acid supply (1.4 million tons annually) [19,20].

The need for efficient genetic tools is paramount to advance fundamental research and develop optimized *Aspergillus* cell factories for diverse applications [21]. Therefore, this review compares the strengths and limitations of conventional gene editing methods versus CRISPR-Cas9 technology in *Aspergillus* research. It first describes the working principles of CRISPR-Cas9 and its adaptation for use in *Aspergillus*. Subsequently, it provides a comprehensive overview of its applications, including the study of pathogenic mechanisms, the disruption of mycotoxin biosynthesis, and metabolic engineering for biomanufacturing. Finally, we discuss ongoing challenges and future directions for CRISPR-Cas9 technology in this field.

## 2. Genetic Editing Techniques in *Aspergillus* Research

**Figure 2:**
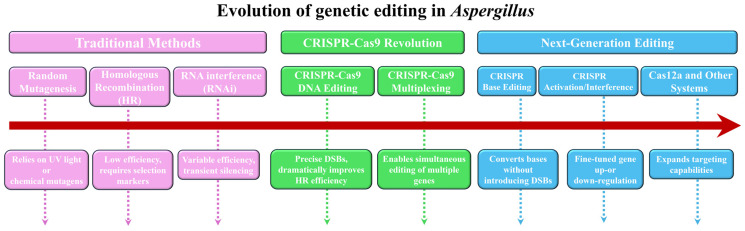
The three development stages of genetic editing techniques in *Aspergillus*.

The genetic engineering of *Aspergillus* species has transitioned from classical random mutagenesis to highly precise and programmable genome editing. This paradigm shift has been instrumental in advancing fundamental research into fungal biology and pathogenicity, as well as facilitating applied biotechnological endeavors. The evolution of these techniques can be broadly categorized into three developmental stages, as outlined in Figure 2.

### 2.1. Limitations of Traditional Genetic Tools in Aspergillus

Before the development of clustered regularly interspaced short palindromic repeats/CRISPR-associated protein 9 (CRISPR-Cas9) technology, genetic manipulation in *Aspergillus* species largely depended on conventional techniques, such as random mutagenesis, RNA interference (RNAi), and homologous recombination (HR). However, these methods pose significant limitations that hinder efficient genetic engineering in fungi, including *A. niger* and *A. oryzae*. Random mutagenesis is the oldest gene editing technology, which employs ultraviolet light or chemical reagents (e.g., N-methyl-N’-nitro-N-nitrosoguanidine (NTG)) to induce random mutations. As a non-targeted approach, it requires subsequent high-throughput screening to identify desired phenotypes, such as increased enzyme yield. While RNAi can silence gene expression at the transcriptional or post-transcriptional level in some *Aspergilli*, it is ineffective in other filamentous fungi due to incomplete or absent RNAi machinery [22]. For instance, species such as *A. nidulans* often exhibit inconsistent or weak RNAi responses due to the absence of core RNAi pathway elements or the presence of RNAi inhibition mechanisms. Even in filamentous fungi with functional RNAi machinery, technical hurdles such as inefficient delivery of double-stranded RNA (dsRNA), transient silencing effects, and off-target interactions often limit its utility in systematic genetic studies or industrial utilization. In contrast, HR-based gene targeting has long served as the standard method for disruption or deletion in *Aspergillus* species. However, this approach is hampered by inherently low efficiency due to poor HR rates and is technically demanding in this genus [22]. Consequently, deleting large gene clusters (e.g., aflatoxin biosynthetic genes in *A. flavus*) requires laborious screening and multiple selection markers [23], and even successful deletions risk leaving residual “cryptic” pathways active, potentially leading to the production of unexpected toxins.

Although HR efficiency can be improved by using strains deficient in non-homologous end joining (NHEJ), the multinucleate nature of *Aspergillus* conidia complicates HR-mediated targeting, as isolating homozygous transformants demands extensive screening [24,25]. In addition, *Aspergillus* genomes harbor a large number of biosynthetic gene clusters (BGCs) for secondary metabolites, yet traditional methods lack the capability to systematically activate, characterize, or manipulate such often-silent clusters. Thus, despite their foundational role, traditional genetic tools are hampered by substantial limitations in efficiency, precision, and applicability. These constraints pose a major bottleneck for the development of engineered strains for biocontrol or industrial biotechnology.

### 2.2. Efficient Editing of CRISPR-Cas9 Technology in Aspergillus

As a powerful and widely adopted genome editing tool, CRISPR-Cas9 technology has been adapted for filamentous fungi through modifications to the Cas protein and single-guide RNA (sgRNA), establishing a versatile system that has significantly promoted genetic manipulation in these organisms [26,27]. The CRISPR-Cas9 system was first established and used to disrupt the *ura5* gene in the filamentous fungus *Trichoderma reesei* [28]. To date, CRISPR-Cas9 systems have been successfully applied to over 40 filamentous fungal species, including *Trichoderma*, *Monascus* [24,25], *Penicillium* [29], *Neurospora* [30,31], *Fusarium* [32] and *Aspergillus* [27,33]. By integrating CRISPR with viral vectors or NHEJ-deficient strains, marker-free edits and high-precision point mutations are achieved, which partially solves the HR challenge caused by low efficiency in filamentous fungi.

The CRISPR-Cas9 system has proven to be a highly effective gene editing tool across diverse *Aspergillus* species. Initial reports as early as 2015 demonstrated its efficacy in *A. nidulans*, *A. niger*, *A. aculeatus*, and *Aspergillus brasiliensis*, where editing efficiency for the *yA* gene (which encodes a key enzyme for green spore pigment biosynthesis) reached up to 90% [27,34]. Since then, further optimization has led to even higher efficiencies. For instance, an optimized CRISPR-Cas9 method utilizing in vitro-assembled Cas9-gRNA ribonucleoprotein (RNP) complexes was developed in *A*. *niger*, achieving 100% targeting efficiency in single-locus genome editing [35,36]. In addition, Chang developed a high-efficiency CRISPR-Cas9 system yielding targeting frequencies exceeding 95% in *A*. *flavus*, which also gave satisfactory gene-targeting efficiencies (>90%) in *A*. *nidulans*, *A*. *fumigatus*, *A*. *terreus*, and *A*. *niger* [37]. In *A. oryzae*, the CRISPR-Cas9 system enables highly efficient genome editing. When combined with selection markers (e.g., based on color or resistance), 100% of the resulting transformants can be obtained with the desired single- or double-gene edits [38]. Meanwhile, Yuan et al. employed two gRNAs for targeting in CRISPR-Cas9 systems and achieved up to 100% editing efficiency for single gene editing in *A. niger* [33]. By targeting a secondary metabolite gene cluster, they also demonstrated that large chromosomal fragments over 100 kb can be efficiently and specifically deleted by a multi-gRNA genome editing system utilizing Cas9 in *A. niger* [33]. Furthermore, in *A. oryzae*, the CRISPR-Cas9 system combined with microhomology-mediated end joining (MMEJ) and single-strand annealing (SSA) repair systems enabled streamlined generation of targeted knock-in transformants, significantly simplifying workflows compared to traditional homology-dependent methods [39]. Collectively, these studies underscore the remarkable efficacy and versatility of the CRISPR-Cas9 system as a genetic engineering tool across the *Aspergillus* genus.

## 3. Working Mechanism of CRISPR-Cas9 System

### 3.1. Type of CRISPR-Cas System

**Figure 3:**
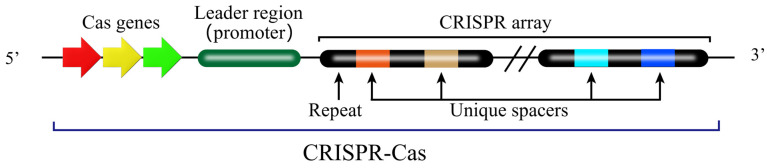
Two key components in the general CRISPR-Cas system locus. The base composition and length of repeat sequences are highly conserved and basically unchanged within the same bacterial species, but they may show slight variations between different bacterial species. The spacer sequences function to anchor target exogenous genes, and thus display significant diversity in their base composition, as these sequences are derived from distinct exogenous gene fragments. Additionally, there is usually leader sequence rich in A-T bases in the upstream of the CRISPR array, which harbors a promoter and is responsible for initiating the transcription of both the repeat and spacer sequences. The schematic employs the following visual code: arrows (red, yellow, green); the leader region (dark green); repeats (black); and unique spacers (orange, gold, light blue, dark blue).

**Figure 4:**
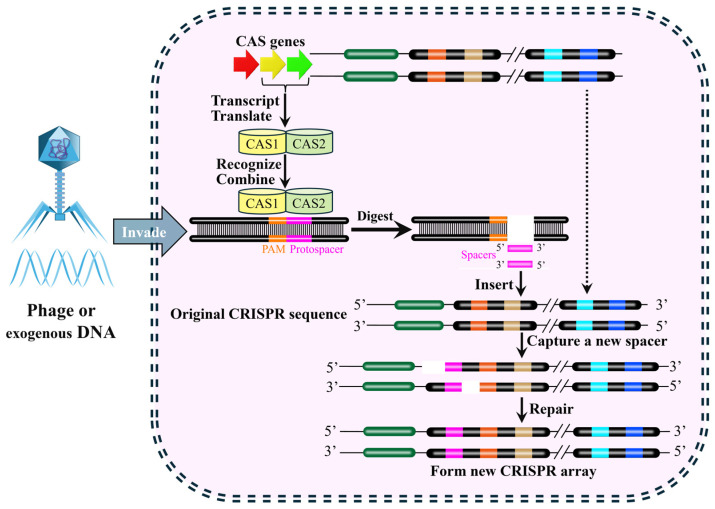
Acquisition of new Spacer sequence in CRISPR-Cas system. When the phages or exogenous genes invade the bacterial, the protospacer in the genome will be recognized and cleaved by Cas-associated proteins in CRISPR-Cas system and inserted into middle of the leader sequence and an adjacent repeat to form a new spacer. This process establishes adaptive immunity, enabling the CRISPR-Cas system to recognize and cleave the same exogenous genome during subsequent invasions. The schematic shows: foreign DNA invasion (blue arrow); key steps (solid black arrows), including protospacer digestion and excision of a new spacer fragment (pink) for integration into the CRISPR array, as detailed in the inset.

The CRISPR-Cas system functions as an adaptive immune system in numerous bacteria and archaea, providing defense against bacteriophage infection and plasmid transfer [40]. The CRISPR-Cas system comprises two key components (Figure 3) [40]. One is the Cas (CRISPR-associated) protein complex, which is responsible for the acquisition of new spacer sequences from invading DNA and for the cleavage of foreign genetic material. The other is the CRISPR array, which consists of short, conserved DNA repeats interspersed by variable spacer sequences that are derived from previous invaders. These components work together in a multi-stage immune response. The first stage is adaptation. During the adaptation phase, short fragments of exogenous DNA derived from invading genetic elements (e.g., phage or plasmids) are integrated into the CRISPR repeat-spacer array within the host chromosome as new spacers, which provides a genetic memory of previous infection that confers immunity against future invasions by the same invader (Figure 4) [41,42,43].

The molecular mechanism described above is encoded by a highly diverse set of genetic systems. The ongoing co-evolution of prokaryotes and the viruses underlies the remarkable diversity of CRISPR-Cas systems [44]. CRISPR-Cas systems are categorized into two main classes (Class 1 and Class 2) based on the structure of their effector complexes [45]. These systems are found in approximately 90% of archaea and 50% of bacteria, with Class 1 systems being the most common, representing nearly 90% of all identified CRISPR-Cas systems [46]. Structurally, Class 1 and Class 2 systems are defined by their effector modules: Class 1 systems (including Types I, III, and IV) utilize multi-protein effector complexes to target and cleave nucleic acids, whereas Class 2 systems (including Types II, V, and VI) employ a single large effector protein [44,45]. The adaptation step, which integrates viral sequences into the host genome, is mediated by the highly conserved Cas1 and Cas2 proteins across nearly all types (except Type IV), underscoring a core conserved function. In contrast, the effector modules responsible for target recognition and cleavage exhibit considerable diversity across the six major types (I-VI) of CRISPR-Cas systems, whose key features are summarized in Table 1 [45,47,48,49,50,51].

To date, most researchers have favored Class 2 CRISPR-Cas systems due to their reliance on a single effector protein. Among these, the type II CRISPR-Cas9 system is the most extensively studied and widely applied in genome editing. It utilizes a single DNA endonuclease (Cas9) to recognize double-stranded DNA (dsDNA) and cleave target DNA sequences [45]. This simplicity and programmability have made CRISPR-Cas9 a revolutionary tool, leading to its recognition as the predominant third-generation genome editing technology, succeeding earlier protein-engineering-based platforms such as zinc-finger nucleases (ZFNs) and transcription activator-like effector nucleases (TALENs) [52]. Consequently, it has now largely superseded these more complex methods for a wide range of applications.

### 3.2. Composition and Working Mechanism of CRISPR-Cas9 System

**Figure 5:**
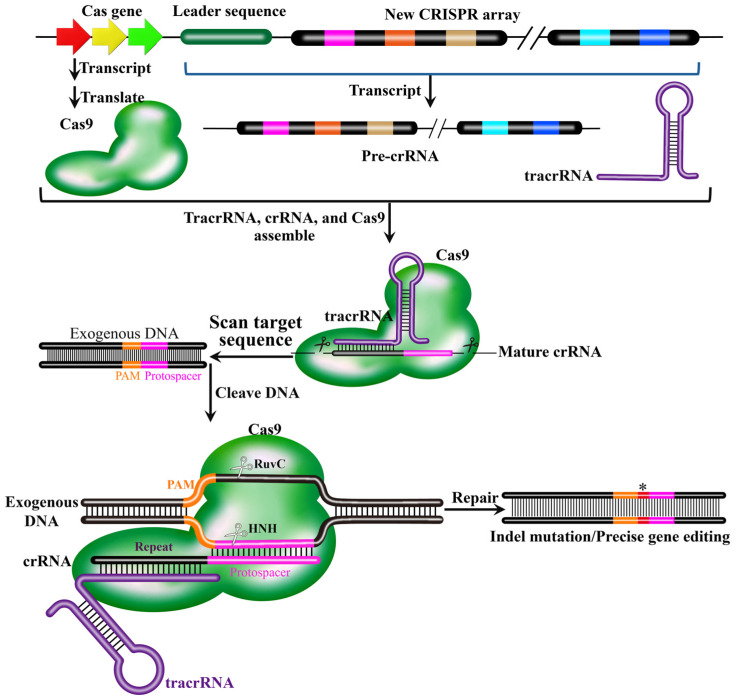
The promoter in the leader sequence initiates the transcription of the downstream CRISPR array. This transcription process is continuous, resulting in a long RNA chain that contains all repeats and spacers in the CRISPR array. This long-chain RNA is called the precursor transcript (Pre-crRNA). Trans-activating crRNA (tracrRNA) is transcribed from a region adjacent to the CRISPR array and Cas9 gene in bacterial genomes. crRNA is complementary to non-coding trans-activating CRISPR RNA (tracrRNA) and then forms a complex with Cas9 protein for target DNA cleavage to generate DSBs. Indel mutation or precise gene editing are achieved during the cellular process of repairing the DSBs. In the schematic, molecular components are color-coded: the Cas9 protein (green irregular shape), tracrRNA (purple ring), pre-crRNA (derive from the new CRISPR array), and invading DNA (black strand with orange PAM and pink protospacer sequences). The red asterisk (*) specifically marks the site of indel mutation generated during the repair of the Cas9-induced double-strand break, located between the PAM and the protospacer.

The functional core of the CRISPR-Cas9 system comprises two elements: the Cas9 endonuclease and a sgRNA. The Cas9 protein, which consists of two endonuclease domains (HNH and RuvC-like) and two RNA-binding domains (REC and PI), is responsible for recognizing and cleaving the target DNA to generate a double-strand break (DSB) immediately upstream of a protospacer adjacent motif (PAM, typically NGG) [53]. The sgRNA is an engineered molecule that mimics the natural tracrRNA:crRNA duplex. In nature, the CRISPR array is transcribed to give rise to a long precursor CRISPR RNA (pre-crRNA), which is subsequently processed into short, mature crRNAs [47]. The 5’ end mature crRNA contains one spacer sequence derived from the invading exogenous DNA, while the 3’ end contains partial repeat sequences flanking the spacer which is critical for stability and Cas protein binding. Trans-activating crRNA (tracrRNA) is a critical non-coding RNA component of type II CRISPR-Cas9 system, transcribed from a region adjacent to the CRISPR array and Cas9 gene in bacterial genomes. The tracrRNA contains a complementary region that base-pairs with the repeat sequence in the pre-crRNA to form a sgRNA [54]. The sgRNA (or the natural tracrRNA: crRNA complex) guides the Cas9 protein to the target DNA via sequence complementarity. Cas9 then cleaves both DNA strands if the target is adjacent to a compatible PAM, thereby generating a DSB [53,55]. During the cellular process of repairing the DSBs, cellular repair mechanisms trigger small base deletions or insertions at the target site through either NHEJ or high-fidelity homology-directed repair (HDR) pathways, generating mutations of frame-shifted nonsense proteins and loss of gene function [56]. The working mechanism of the CRISPR-Cas9 system described is shown in Figure 5.

### 3.3. Functional Features of Cas9 and PAM in CRISPR-Cas9 System

The practical application of CRISPR-Cas9 hinges on two key design elements: the guide RNA and the protospacer adjacent motif (PAM). Regarding the guide RNA, scientists often use engineered sgRNA instead of separate crRNA and tracrRNA. The crRNA and tracrRNA are fused into sgRNA in vitro, simplifying delivery while retaining tracrRNA’s functional elements (e.g., Cas9 binding and structural stabilization). But in the natural system, crRNA and tracrRNA are separate. TracrRNA is required for Cas9 but not necessarily for other CRISPR-associated proteins (e.g., Cas12 or Cas13). Regarding the PAM, the recognition and acquisition of spacers by Cas9 proteins depend on the PAM sequence located downstream of the target DNA. The PAM is a short, conserved sequence (2-6 bp) located adjacent to the 3’ end of the crRNA-targeted DNA sequence (protospacer) on the invading DNA, which plays an essential role in target DNA selection and cleavage in the CRISPR-Cas9 systems [57]. Additionally, this PAM plays a critical role in the in vitro design of CRISPR-Cas9 system. Different Cas9 proteins recognize distinct PAM sequences, influencing their utility in fungal genome editing.

Despite being the most widely used effector protein due to its high efficiency in generating DSBs [58,59], the Cas9 from *Streptococcus pyogenes* (SpCas9) has three principal limitations that constrain its application: First, its PAM is NGG, which requires the target sequence to contain two consecutive guanines (GG) for DSB formation, thereby restricting its targeting scope in AT-rich genomic regions. Second, the relatively large size of SpCas9 (1368 amino acids) complicates its delivery via viral vectors with limited packaging capacity. Third, SpCas9 is prone to off-target effects, leading to DSBs at unintended genomic loci. To address these challenges, several engineered strategies have been developed. For instance, high-fidelity variants such as SpCas9-HF have been created through coding sequence modifications to reduce off-target effects [60]. In addition, to overcome the PAM constraint, SpCas9 has been engineered to recognize alternative PAMs. The resulting variants are often named by abbreviated codes (e.g., VQR, EQR, VRER) that reflect the key amino acid substitutions responsible for their altered PAM specificity [61]. Another engineered derivative of SpCas9 is the nickase Cas9 (nCas9), created by inactivating one of the two nuclease domains in SpCas9. This variant induces single-strand breaks and, when used in combination with two gRNAs, can generate targeted deletions or other genomic modifications while reducing off-target effects [62,63]. To address the large size of SpCas9, Cas9 orthologs from other organisms have been utilized. For instance, *Staphylococcus aureus* Cas9 (SaCas9) is notably smaller and recognizes a different PAM sequence [64,65]. A summary of commonly used Cas9 proteins and their respective PAM sequences is provided in Table 2 [58,59,64,65,66,67,68,69].

## 4. Key Applications of CRISPR-Cas9 Technology in *Aspergillus*

### 4.1. Elucidating Pathogenicity and Drug Resistance

Certain *Aspergillus* species, though generally harmless to healthy individuals, can pose significant health risks to immunocompromised hosts. As the most prevalent airborne fungal pathogen, invasive aspergillosis caused by *A*. *fumigatus* primarily occurs in immunocompromised patients and has dramatically increased since the early 2000s [70]. However, research into its pathogenic mechanisms has long been hindered by its inherently low efficiency of HR, which favors the NHEJ pathway. Prior to the advent of CRISPR-Cas9, researchers relied on labor-intensive strategies to improve HR or work within its constraints, such as the use of recyclable selectable markers or the development of dominant bidirectional selection systems (e.g., Krappmann et al., 2005) [71]. The emergence of CRISPR-Cas9 genome editing has revolutionized this field. By providing a powerful and precise tool to directly overcome this barrier, it now enables detailed functional studies of virulence factors in *A. fumigatus.*

CRISPR-Cas9 has been used to identify genes involved in stress response, drug resistance, and host immune evasion, providing insights into developing new antifungal therapies [72]. Fuller et al. first demonstrated the feasibility and high efficiency (25–53%) of CRISPR-Cas9-mediated gene editing in *A. fumigatus* by targeting disruption of *pksP*, a polyketide synthase gene essential for melanin biosynthesis [73]. Zhang et al. subsequently developed a highly efficient CRISPR-Cas9 system that leverages the MMEJ pathway, using a 35 bp microhomology arm to achieve precise in-frame integrations with 95–100% accuracy [74]. Utilizing this MMEJ-mediated CRISPR-Cas9 system, they achieved precise integration of an exogenous GFP tag and performed editing at multiple genomic loci, including the endogenous *pksP* and *cnaA* genes. A major breakthrough in *A. fumigatus* involved the combination of CRISPR-Cas9 with endogenous counter-selectable markers (e.g., *fcyB*, *cntA*, *azgA*). This approach allows for efficient, marker-free allelic replacement and facilitates the simultaneous integration of multiple constructs (e.g., reporter and resistance genes) in a single transformation, vastly improving the throughput of complex genetic studies [75]. Collectively, these technological breakthroughs have not only overcome historical limitations in fungal genetic engineering but also established a solid framework for systematic study of virulence factors, antifungal resistance, and secondary metabolite pathways in *A*. *fumigatus*.

The CRISPR-Cas9 technology has been used to elucidate antifungal resistance mechanisms in *Aspergillus* [76]. For example, in *A. fumigatus*, CRISPR-Cas9 has been instrumental in linking specific mutations to altered drug-target interactions. Triazole antifungals, such as isavuconazole (FDA-approved in 2015 for invasive aspergillosis and mucormycosis), remain frontline therapies. Their target, the enzyme encoded by the *Cyp51A* gene, is a lanosterol 14α-demethylase essential for ergosterol biosynthesis, a key component of the fungal cell membrane [77,78]. Recent studies leveraging CRISPR-Cas9 have elucidated the mechanistic roles of Cyp51A, Cyp51B, and Hmg1 (hydroxymethylglutaryl-CoA reductase) in resistance. For instance, RNP-based CRISPR-Cas9 systems enable precise introduction of resistance-conferring mutations in these genes, revealing their synergistic contributions to triazole tolerance in *A. fumigatus* [79,80]. Furthermore, an in vitro-assembled CRISPR-Cas9 system streamlined the integration of mutations in *cyp51A*, *cyp51B* and *hmg1* in *A. fumigatus*, providing robust models to dissect resistance evolution [81].

Beyond elucidating established resistance mechanisms, CRISPR-Cas9 has also accelerated de novo antifungal drug discovery through functional genomics. A CRISPR-Cas9 RNP screen targeting protein kinase genes in *A. fumigatus* identified several kinases critical for fungal survival under echinocandin stress [82]. Importantly, these kinases are also required for both hyphal septation and the ability of *A. fumigatus* to invade lung tissue. The versatility of CRISPR-Cas9 extends beyond resistance studies. With the potential to seamlessly introduce genes conferring resistance to additional existing and novel antifungals, toxic metals, and environmental stressors, the CRISPR-Cas9 system has become an indispensable tool to explore the mechanisms of fungal adaptation. Such advances not only enhance our understanding of *A*. *fumigatus* biology, but also provide strategies for avoiding drug resistance and designing the next generation of antifungal drugs.

### 4.2. Disruption of Toxin Biosynthesis in Aspergillus

CRISPR-Cas9 technology has revolutionized genetic engineering in fungi, offering unprecedented precision for developing biocontrol solutions against toxigenic *Aspergillus* species. *A. flavus* produces hepatocarcinogenic aflatoxin B1, recognized as one of the most potent hepatocarcinogens among natural compounds. *A. flavus* can grow in various food crops, and preharvest aflatoxin contamination of crops is a complex problem. The most cost-effective approach to control preharvest aflatoxin contamination of crops is biological control, which employs non-aflatoxigenic *A. flavus* strains with defective aflatoxin gene clusters to outcompete field toxigenic *A. flavus* populations [83]. Beyond aflatoxin B1, *A. flavus* produces additional toxic secondary metabolites, including cyclopiazonic acid, ustiloxin B, and aflatrem. This multiplicity of toxins necessitates comprehensive genetic modifications to ensure biocontrol strain safety. Genome editing can be utilized to obtain non-aflatoxigenic *A. flavus* strains, typically by generating loss-of-function point mutations in key biosynthetic genes (e.g., *pksA*, *dmtA*) or by deleting segments of the aflatoxin biosynthetic gene cluster [84].A significant study by the United States Department of Agriculture utilized a dual CRISPR-Cas9 system to create large chromosomal segment deletions (201–301 kb) encompassing the gene clusters for aflatoxin, cyclopiazonic acid, and ustiloxin B in *A*. *flavus* [23]. This strategy achieved high deletion efficiency (66.6% to 85.6%) using short 60-nucleotide donor DNA and a pigment-based screening system, demonstrating an effective method for generating biocontrol strains devoid of multiple harmful metabolites.

This strategy of multi-toxin cluster deletion has also been applied to *A. oryzae*. Wild-type strains were engineered to remove gene clusters for aflatoxin, cyclopiazonic acid, 3-nitropropionic acid, and penicillin G. This was achieved through multiple rounds of gene editing, resulting in strains devoid of these mycotoxins even under stress conditions [85]. Furthermore, the versatility of CRISPR-Cas9 for pathway manipulation is underscored by its application in *A. fumigatus*. Although the direct disruption of the gliotoxin pathway has not been extensively reported, CRISPR-Cas9 has been successfully applied in *A. fumigatus* to reconstitute biosynthetic pathways for metabolites such as chetomin [77]. This successful reconstitution demonstrates the platform’s general capability for precise pathway manipulation, paving the way for its application in the targeted disruption of virulence-associated pathways such as gliotoxin biosynthesis.

### 4.3. Metabolic Engineering for Bioproduction

**Figure 6:**
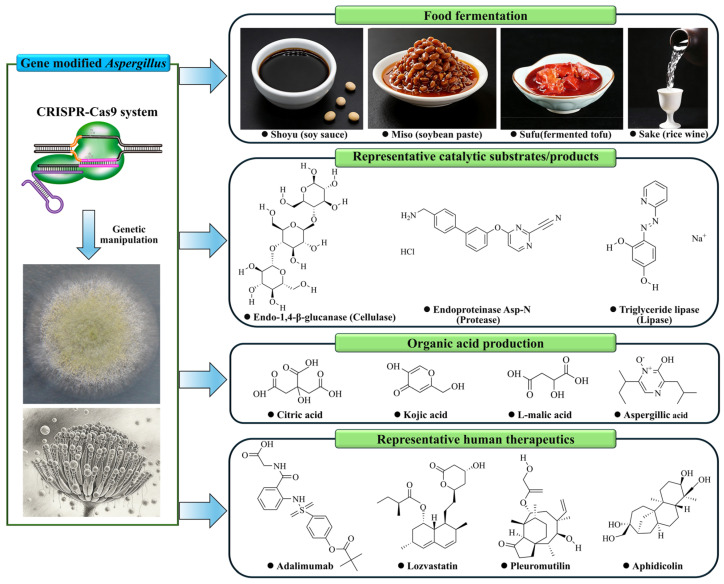
CRISPR-Cas9 engineering expands the application scope of *Aspergillus* species. This schematic uses representative molecular structures to visualize key product categories that are now accessible or being explored through advanced metabolic engineering of *Aspergillus* species. The molecules are grouped into four major application sectors: traditional fermentation and food, industrial enzymes (exemplified by endo-1,4-β-glucanase, endoproteinase Asp-N, and triacylglycerol lipase), organic acids (e.g., citric acid), and pharmaceuticals (including biologic drugs like the monoclonal antibody adalimumab, and small-molecule drugs such as the statin lovastatin, the antibiotic pleuromutilin, and the DNA synthesis inhibitor aphidicolin). This compilation conceptualizes how precision genome editing can reprogram these fungi into versatile platforms for diverse biomanufacturing goals.

The fungal genus Aspergillus, particularly species like A. oryzae and A. niger, has long been used for food fermentation and valued in biotechnology as efficient cell factories for the production of enzymes, organic acids, and secondary metabolites [19,86,87]. However, the advent of the CRISPR-Cas9 genome editing system, as a multifunctional technology, has revolutionized metabolic engineering in these organisms by enabling precise, multiplexed genetic manipulations that were previously challenging or impossible. Thereby, this has dramatically accelerated the optimization of industrial Aspergillus strains for enhanced bioproduction [24]. The broad utility of this technology is visualized in Figure 6, where representative molecules exemplify key application areas enabled by CRISPR-Cas9 engineering in Aspergillus: traditional food fermentation, industrial enzyme production, organic acid biosynthesis, and the burgeoning field of pharmaceutical development.

#### 4.3.1. Enhancing Industrial Enzyme and Protein Production

A major application of CRISPR-Cas9 in *Aspergillus* is the construction of tailored cell factories for efficient protein and enzyme production, extensively engineering these fungi into high-yielding platforms. A foundational strategy involves knocking out genes encoding native secreted proteases to prevent the degradation of target proteins and thereby increase yields. Beyond gene disruption, CRISPR-Cas9 technology is particularly powerful for precise genomic integration. For instance, in *A*. *oryzae*, CRISPR-Cas9 has been used to disrupt lipase genes of *AoTgla* and *AoTglb* to alter lipid metabolism and to integrate multiple heterologous lipase genes into different genetic loci in *A. oryzae* [38,88]. CRISPR-Cas9 was also used to reprogram the transcriptional regulation of the *acv* gene, which encodes ACV synthetase (ACVS), to develop a platform for producing bioactive oligopeptides (NRPs) in *A. oryzae* [89]. In *A. niger*, significant advancements were achieved by developing an improved CRISPR-Cas9 homology-directed repair (CRISPR-HDR) system, which enabled the precise integration of a glucose oxidase gene (*GoxC*) into the *amyA* and *glaA* loci, resulting in a fourfold increase in enzyme activity [90]. Based on this CRISPR-HDR system, CRISPR-based multiplex integration toolkit was constructed in *A. niger*, achieving 100% editing efficiency when simultaneously integrating the xylanase gene *xynA* into three target loci (the β-glucosidase gene *bgl*, the amylase gene *amyA*, and the acid amylase gene *ammA*) [91]. This multiple integration toolkit also successfully enhanced the expression of endogenous pectinase *pelA* and *Candida antarctica* lipase B (CALB) [91]. Furthermore, an engineered *A. niger* strain with high lipase activity was successfully isolated by employing the CRISPR-Cas9 system to integrate CALB into high-expression loci of *glaA* and *amyA*, while simultaneously knocking out the host’s highly expressed protein genes of *pepA*, *aglU*, and *bglA* [92]. This multi-faceted engineering approach yielded an *A. niger* strain producing 10.21 mg/mL of CALB protein with activity reaching 17.84 U/mL. The application extends to other species, such as *A. fumigatus*, where CRISPR-Cas9-mediated targeted knock-in of the cellulase gene *eglA* from *A. niger* led to a 40% increase in enhanced endoglucanase activity, highlighting its potential in enzyme production [93]. This is the first report of heterologous cellulase production in filamentous fungi using CRISPR-Cas9 technology.

Beyond protein expression, the CRISPR-Cas9 system was used to delete the *Aooch1* gene, which encodes a key enzyme in the hyper-mannosylation process in *A. oryzae,* in order to investigate the binding ability of antibody for FcγRIIIa [12]. In addition, CRISPR-Cas9 technology was employed to examine the association between protein/organic acid fermentation and macromorphology in *A. niger* by placing the titratable Tet-on expression system upstream of *ageB*, *secG*, and *geaB*, which led to improved titres [94]. Together, this study showed that the integration of CRISPR-Cas9 with the Tet-on expression system provides a novel strategy to enhance protein yield. Using a CRISPR-Cas9-mediated multicopy integration strategy, the trehalases TreM and MthT, which catalyze the hydrolysis of the non-reducing disaccharide trehalose, were heterologously expressed in *A. niger*, reaching a yield of 1943.06 U/mL and 1698.83 U/mL, respectively [95,96]. Furthermore, CRISPR-Cas9 has been used to modify protein secretion pathways to address productivity barriers in *A. nidulans*, including post-translational modifications such as protein N-glycosylation [97]. In that study, a double knockout of the *algC* and *algI* genes via CRISPR-Cas9 altered the glycosylation pattern of a recombinant β-xylosidase (BxlB), resulting in a 1.5-fold improvement in secretion and an increase in specific activity. These collective successes underscore the transformative potential of CRISPR-Cas9 in optimizing *Aspergillus* as a cell factory.

#### 4.3.2. Engineering for Organic Acid Production

The precision of CRISPR-Cas9 genome editing has been pivotal in engineering *Aspergillus* species for enhanced organic acid production. This section reviews key advances in this area, including the development of metabolic engineering strategies for citric acid and the application of CRISPR-Cas9 systems for genome editing in *A. niger*. In particular, *A. niger* dominates the $2.8 billion/year citric acid market via submerged fermentation [20]. A significant breakthrough was the development of a highly efficient CRISPR-Cas9 system for *A*. *niger* by researchers at the Tianjin Institute of Industrial Biotechnology [98]. They addressed the challenge of expressing sgRNA in fungi by using the 5S rRNA gene as a promoter to drive sgRNA expression. This system simplifies genetic manipulations, allowing for gene knock-ins with short 40 bp homology arms and even large DNA deletions up to 48 kb, thereby providing a robust tool for extensive metabolic engineering. As the key industrial workhorse for organic acids, *A. niger* has been a primary target for CRISPR-Cas9-enabled precise gene knockouts, promoter engineering, and multi-gene integrations to optimize metabolic pathways. For example, it allows for rational strain improvement by precisely eliminating genes responsible for major byproducts and gluconic acid, thereby channeling more carbon flux towards citrate. For instance, overexpressing key glycolytic enzymes like phosphofructokinase (*PfkA*) and pyruvate kinase (*PkiA*) can increase glycolytic flux, while strategies like knocking out the citrate synthase gene (*gltA*) can redirect carbon flow away from byproducts and towards citric acid products [99]. A 2025 study used CRISPR-Cas9-mediated Tet-on inducible promoter replacement to titrate the expression of *flbE*, a key developmental regulator gene, in two industrial *A*. *niger strains*. The researchers discovered that repressing *flbE* in a citric acid-producing strain (D353.8) resulted in a significantly increased citric acid production by 22.7% [100].

Beyond citric acid, the platform is being applied to other valuable organic acids. For example, CRISPR-Cas9 together with sgRNA synthesized in vitro was used to successfully delete multiple genes involved in galactaric acid catabolism for the efficient production of galactaric acid in *A. niger* [101]. This study demonstrated a successful early application of CRISPR-Cas9 for galactaric acid production in *A. niger*. Subsequently, Kuivanen et al. used CRISPR-Cas9 system to effectively delete the *gluD* gene of D-glucuronic acid catabolism in *A. niger*, resulting in the accumulation of 2-keto-L-gulonate in the liquid cultivation [102]. Furthermore, an optimized CRISPR-Cas9 method utilizing in vitro-assembled Cas9-gRNA RNP complexes was developed, achieving 100% targeting efficiency in single-locus genome editing in *A*. *niger* [35,36]. This optimized CRISPR-Cas9 method has also been proven to be suitable for multiplexed genome editing with two or three genomic targets in metabolic engineering application, resulting in improved production of galactarate in *A. niger* [35]. In another study, an RNP-based CRISPR-Cas9 system has been successfully used to disrupt two genes involved in the production of gluconic acid and oxalic acid in *A. niger,* which significantly improved the succinic acid production [103].

In addition, a convenient and efficient double-gene editing system based on CRISPR-Cas9 and Cre-loxP was developed in *A. nidulans* to investigate its potential as a host for L-malic acid synthesis [104]. Using this developed gene editing system, five genes (encoding *Pyc*, pyruvate carboxylase; *OahA*, oxaloacetate acetylhydrolase; *MdhC*, malate dehydrogenase; *DctA*, C4-dicarboxylic acid transporter; and *CexA*, citric acid transporter) were successfully deleted or overexpressed. These modifications increased the L-malic acid production by approximately 9.6-fold compared with the original unedited strain. Additionally, Zhang et al. employed CRISPR-Cas9 system to replace the *fumA* promoter with a doxycycline-induced promoter Tet (Tet-on/off system) in a high L-malic acid-producing strain RG0095, indicating that *fumA* is an essential gene and contributes to the accumulation of L-malic acid in *A. niger* [105].

Furthermore, CRISPR-Cas9 technology was also used to elucidate and enhance kojic acid biosynthesis in *A. oryzae* by targeting various genes involved in its regulatory and metabolic pathways. For instance, CRISPR-Cas9-mediated disruption of the glycerol dehydrogenase gene *AoGld3* revealed that its deletion reduces kojic acid production, and downregulates the expression of the biosynthetic genes *kojA* and *kojR* [106]. In another study, Li et al. employed an AMA1-based CRISPR-Cas9 system to generate single mutants of *kojA*, *kojR*, and *kojT*, thereby establishing a platform for multiplex gene editing in *A. oryzae* [107]. Similarly, the CRISPR-Cas9 system was utilized to delete *Aokap5* and *AoKap7* in *A. oryzae. AoKap7* is a C_2_H_2_-type zinc-finger protein involved in growth and kojic acid production. Their deletion resulted in reduced kojic acid production and downregulated expression of kojic acid biosynthesis genes *kojA* [38,108]. More recently, CRISPR-mediated knockout of the *Aokap9* gene unveiled a novel regulatory pathway (*AoZFA*-*LaeA*-*KojR*) for kojic acid synthesis, leading to a significant increase in kojic acid production [109]. Collectively, these findings provide a deeper understanding of the regulatory network controlling kojic acid biosynthesis.

#### 4.3.3. Reprogramming Natural Product Biosynthetic Pathways

The CRISPR-Cas9 system further demonstrates its versatility in reprogramming natural product biosynthetic pathways in diverse *Aspergillus* species, enabling both targeted pathway engineering and activation of silent gene clusters. Specifically, CRISPR-Cas9 has proven valuable for reviving silent biosynthetic pathways. Weber et al. successfully restored the biosynthesis of trypacidin in a nonproducing *A. fumigatus* strain by using the CRISPR-Cas9 system to functionally reconstitute *tynC*, a polyketide synthase-encoding gene in the trypacidin pathway [110]. In *A. terreus*, optimized CRISPR-Cas9 protocols have enabled precise editing (knockout and knock-in) of key genes (*lovB*, *lovF*, *lovR*) in the lovastatin biosynthesis pathway, showcasing its power for metabolic engineering in industrial settings [111]. Furthermore, to reduce the cumbersome hydrolysis of lovastatin to produce Monacolin J in *A. terreus*, key genes (*lovB*, *lovC*, *lovG*, and *lovA*) involved in the Monacolin J biosynthetic pathway were heterologously integrated into the *A. niger* genome with strong promoters and suitable integration sites using CRISPR-Cas9 homology-directed recombination, and the yield of Monacolin J reached 92.90 mg/L [112]. In recent studies, CRISPR-Cas9 genome editing was used to engineer *A. oryzae* for heterologous production of terpenoids. Through 13 targeted genetic modifications, researchers achieved enhanced production of pleuromutilin, aphidicolin, and ophiobolin C compared to the unmodified *A. oryzae* strain [113].

CRISPR-Cas9-mediated transcriptional activation has also been used to accelerate the discovery of genome-guided bioactive natural products in *A. nidulans.* For instance, using the established CRISPR strategy, increased production of the compound microperfuranone was achieved by targeting the native nonribosomal peptide synthetase-like (NRPS-like) gene *micA* in *A. nidulans* [114]. In a more recent advancement, a CRISPR-Cas9 cytidine base editor (CBE) combined with a multiplexed sgRNA library enabled the simultaneous inactivation of 46 natural product biosynthetic gene clusters in *A. nidulans*, which reduced competing byproduct synthesis and increased the yield of the antifungal compound echinocandin B by 2.3-fold [115].

The versatility of CRISPR-Cas9 extends to precise transcriptional activation using a catalytically dead Cas9 (dCas9). This was demonstrated in *A*. *niger*, where dCas9 fused to a histone acetyltransferase (p300) was targeted to the promoter of a specific secondary metabolite gene. This recruitment led to substantially increased gene expression and a dramatic 12-fold enhancement in fumonisin B2 production [116,117]. Additionally, CRISPR-Cas9 technology has also been applied to *A. oryzae* to elevate intracellular levels of nutraceuticals such as ergothioneine and flavor/color molecules like heme in the edible biomass. Using a modular toolkit that incorporated CRISPR-Cas9, researchers engineered the fungus to express an optimized heme biosynthesis pathway, resulting in a 4-fold increase in intracellular heme. This imparted a meat-like reddish hue to the fungal biomass, enhancing its potential as an animal meat analog [118].

## 5. Discussion and Future Perspectives

The advent of the CRISPR-Cas9 system, enabling precise, efficient, and multiplexed genome editing, has overcome the limitations of traditional methods and dramatically accelerated research and industrial breeding in *Aspergillus.* The CRISPR-Cas9 system has been used on a variety of pathogenic and industrially important *Aspergillus* species, including *A*. *fumigatus*, *A. flavus*, *A. terreus, A*. *oryzae*, *A*. *niger*, and *A*. *nidulans*. By targeting *Aspergillus*-specific genes involved in metabolic pathways, developmental regulation, or stress responses, researchers can elucidate gene function and regulatory mechanisms with improved precision. Specifically, it is used to optimize fungal cell factories for sustainable production, to elucidate the pathogenicity of plant pathogens, and to silence virulence factors in human pathogens. Its versatility, extending from gene knockout to precise knock-in and base editing, directly accelerates the discovery of novel bioactive compounds and enables the tailored improvement in strains for industrial adaptability. However, the application of CRISPR-Cas9 systems in filamentous fungal factories, while established in principle, is far from being industrially mature. Current research frontiers are actively addressing these gaps, as exemplified by the recent development of a visual, multiplex integration toolkit for *A. niger*, which achieved 100% editing efficiency at single loci and enabled one-step, marker-free integration of expression cassettes into three genomic loci, boosting enzyme yield by nearly 50% [91].

Looking beyond these foundational efforts, the ultimate translation of engineered strains into industrial-scale production presents a distinct set of challenges. The industrial application of CRISPR-Cas9-engineered *Aspergillus* strains faces significant hurdles, primarily in ensuring long-term genetic stability for cost-effective bioprocessing without antibiotic selection. This necessitates stable genomic integration of engineered pathways, a fundamental requirement for reliable industrial bioproduction [119]. Furthermore, advanced synthetic biology strategies, such as the balanced-lethal systems used to maintain plasmid stability in engineered microbial therapeutics, provide a valuable blueprint for enhancing the robustness of fungal cell factories [120]. As these enabling technologies mature alongside a deeper understanding of fungal physiology, the precision, efficiency, and ultimately the industrial robustness of engineered *Aspergillus* strains are poised for significant advancement. Notably, species such as *A. oryzae* and *A. niger,* which have a long history of safe use in the food industry as GRAS organisms [121], are particularly promising chassis. These species are poised to serve as superior cell factories, making remarkable strides in the production of beneficial secondary metabolites and secreted proteins.

To fully realize this potential, future advancements in CRISPR-Cas9 systems for filamentous fungi must focus on overcoming several key barriers, such as low transformation efficiency and CRISPR-associated cytotoxicity. These barriers, along with the broader challenges and methodological considerations for applying CRISPR-Cas9 in filamentous fungi, have been systematically reviewed [122]. The general strategies to overcome them—including optimizing expression systems, delivery methods, and Cas9 variants—are well outlined in CRISPR tool development for unicellular fungi like *yeast*, providing a crucial conceptual framework for adaptation in filamentous species [123]. For example, the efficacy of CRISPR-Cas9 is closely related to the recognition of compatible PAMs, which restrict editable genomic loci. While the classical NGG PAM is dominant in current applications, exploring novel Cas9 variants or orthologs (e.g., SaCas9, FnCas9, or engineered Cas9-NG) with a wider range of PAM recognition (e.g., NRN, NYN, or relaxed motifs) could unlock previously inaccessible regions in *Aspergillus*. The principle and high efficiency of this approach have been robustly demonstrated in model systems such as yeast, where the “GTR 2.0” system utilizing SpCas9-NG achieved near 100% editing efficiency across all NGN PAM sequences [124], providing a strong technical blueprint for adaptation in *Aspergillus.* Moving forward, the combination of the CRISPR-Cas9 system with high-throughput screening methods is a promising research direction to establish a general editing system that is not limited to specific fungal species. Furthermore, coupling CRISPR-Cas9 with synthetic biology and metabolic engineering can unlock the full potential of fungal systems for diverse applications, including the discovery of novel bioactive compounds and the optimized production of valuable metabolites and proteins. By refining these synergies, CRISPR-Cas9 is poised to bridge the translation gap between laboratory discoveries and real-world biotechnological solutions.

## 6. Conclusions

The introduction of CRISPR-Cas9 technology marks a fundamental transformation in the genetic manipulation of *Aspergillus* fungi. As this review has detailed, the system has overcome the limitations of conventional methods. It now acts as a pivotal link between fundamental fungal biology and applied biotechnology, enabling precise investigations into pathogenicity, the reprogramming of metabolic pathways, and the development of efficient cell factories.

To fully realize this potential, the field must now shift its focus from proving technical feasibility to achieving robust and scalable industrial applications. The critical steps forward involve overcoming persistent bottlenecks in editing efficiency and genetic stability, followed by seamless integration with synthetic biology platforms. Success in this endeavor will cement engineered *Aspergillus* strains, with a special emphasis on those possessing established safety profiles, as versatile and sustainable cell factories for future biomanufacturing.

## Figures and Tables

**Table 1:** The key features of six CRISPR-Cas systems.

System Type	Signature Protein(s)	Target Molecule	Key Features	PAM Requirement	Cleavage Mechanism	References
Type I	Cas3 (helicase-nuclease)	DNA	Features a multi-subunit Cascade complex for surveillance and the Cas3 protein for processive DNA degradation.	Yes	Cas3 makes long-range deletions.	[45,47]
Type II	Cas9	DNA	The most well-studied system. Uses a single Cas9 protein and a sgRNA. Cuts DNA, creating double-strand breaks. Diverse subtypes (II-A, B, C, D) with varying sizes and efficiencies exist.	Yes (e.g., SpCas9: NGG)	HNH and RuvC domains cut complementary and non-complementary strands, often resulting in blunt ends.	[45,48]
Type III	Cas10	RNA/DNA	Complex systems that can target RNA. Some subtypes exhibit collateral, non-specific cleavage of RNA or DNA upon target recognition.	-	Involves the Cas10 protein.	[45,49]
Type IV	Unknown	DNA	Functionally less characterized. Believed to play a role in plasmid interference.	-	-	[45]
Type V	Cas12 (e.g., Cas12a/Cpf1)	DNA	Effectors like Cas12 are often smaller than Cas9. They originate from TnpB transposons, an evolutionary path revealed by the discovery of “TranC” intermediates. Cas12a.	Yes	A single RuvC domain cuts both DNA strands, creating staggered ends with overhangs.	[45,50]
Type VI	Cas13	RNA	Targets and cleaves RNA molecules. Upon activation, some Cas13 proteins exhibit non-specific “collateral” cleavage of nearby RNAs, which has been leveraged for sensitive diagnostic tools like SHERLOCK.	-	-	[45,51]

**Table 2:** The structural characteristics of different PAM sequences in the CRISPR-Cas9 system.

Cas9 Source	Cas9 Type	Cas9 Name	PAM Sequence	Characteristics	References
*Streptococcus pyogenes*	Natural Cas9 orthologs	SpCas9	NGG	Widely used; 3′-NGG on the non-target strand.	[58,59]
*Staphylococcus aureus*	Natural Cas9 orthologs	SaCas9	NNGRRT (R=A or G)	Original PAM, restrictive.	[64,65]
*Campylobacter jejuni*	Natural Cas9 orthologs	CjCas9	NNNNACAC	Ultra-compact; useful for tight spaces.	[66]
*Streptococcus equinus HC5*	Natural Cas9 orthologs	SeHCas9	NAG, NKG (K=G or T), NAW (W=A or T)	Similar to SpCas9 but harbored a different RxQ PAM-binding motif.	[59]
*Streptococcus pyogenes*	Engineered variants	xCas9	NG, GAA, GAT	Broadened PAM recognition via directed evolution.	[58]
*Campylobacter jejuni*	Chimeric variants	Hsp1-Hsp2Cas9	N4CY (Y=C or T)	Few off-targets compared to SpCas9.	[67]
*Streptococcus equinus HC5*	Engineered variants	SeHdCas9-RR	NNG, NNN	Achieved PAM-free editing in bacterial systems.	[68]
*Streptococcus canis*	engineered variants	ScCas9++	NNG	With high specificity and minimal off-target effects.	[69]

N = any nucleotide.

## Data Availability

Data sharing is not applicable to this article as no new datasets were generated or analyzed during this study. This review is based on previously published studies, all of which are cited in the reference list.
